# Supplementary material for: Earliest evidence for fruit consumption and potential seed dispersal by birds
Source: eLife. 2022 Aug 16;11:e74751. doi: 10.7554/eLife.74751 (PMC9381037; doi:10.7554/eLife.74751)
Supplement: Figure 2—source data 1. [file elife-74751-fig2-data1.docx]

| **Landmark name** | **Type** | **Landmark description** |
| --- | --- | --- |
| Upper jaw beak tip | Landmark | Rostral-most point on the bill tip of the upper jaw. |
| Craniofacial hinge | Landmark | Mid-point of the craniofacial hinge, the meeting point between the upper jaw and cranium. |
| Jugal bar start | Landmark | Caudal-most point of the jugal bar at the articulation with the quadrate. |
| Jugal bar end | Landmark | Rostral-most point of the jugal bar that connects to the upper jaw. |
| Jugal bar | Semi-landmark | Starting at the ‘jugal bar start’, sampling the jugal bar in a caudal to rostral direction, and ending at the ‘jugal bar end.’ |
| Upper jaw ventral bar start | Landmark | The caudal-most point of the upper jaw ventral bar. In most specimens, this landmark has the same position as the ‘jugal bar end’ landmark, but for some it will be located further caudoventral due to extension of the upper jaw. |
| Upper jaw ventral bar | Semi-landmark | Starting at the ‘upper jaw ventral bar start’, sampling along the upper jaw ventral bar in a caudal to rostral direction, and ending at the ‘upper jaw beak tip.’ |
| Upper jaw dorsal bar | Semi-landmark | Starting at the ‘upper jaw beak tip’, following the upper jaw dorsal bar from rostral to caudal direction, and ending at the ‘craniofacial hinge.’ |
| Nasal aperture | Semi-landmark | Starting from the point of maximum curvature on the caudal end of the nasal aperture, sampling along the dorsal border of the aperture in a caudal to rostral direction until the point of maximum curvature on the rostral end, and across the ventral border in a rostral to caudal direction, just rostral to the first semi-landmark. |
| Orbit | Semi-landmark | Starting from the tip of the processus postorbitalis, following the orbital crest in a caudal to rostral direction, sampling along the edge of the os lacrimale and stopping at its most ventrolateral point. |

Figure 2 - Source data 1. Descriptions of cranial and upper jaw landmarks and semi-landmarks (following Bjarnason and Benson, 2021).
