# Supplementary material for: Earliest evidence for fruit consumption and potential seed dispersal by birds
Source: eLife. 2022 Aug 16;11:e74751. doi: 10.7554/eLife.74751 (PMC9381037; doi:10.7554/eLife.74751)
Supplement: Figure 2—source data 2. [file elife-74751-fig2-data2.docx]

| **Landmark name** | **Type** | **Landmark description** |
| --- | --- | --- |
| Craniodorsal dentary | Landmark | Tip of the craniodorsal dentary, on the ventral surface. |
| Mandibular symphasis | Semi-landmark | Starting at the ‘anterodorsal dentary’ on the ventral surface of the mandibular symphasis, sampling in a cranial to caudal direction, ending at the caudal-most position. |
| Dorsal mandible | Semi-landmark | Starting at the end of the ‘mandibular symphasis’, sampling in a cranial to caudal direction along the mid-line of the ventral surface of the mandible, ending at the caudoventral peak of the caudal lower mandible. |
| Ventral mandible | Semi-landmark | Starting at the beginning of the ‘mandibular symphasis’, sampling in a cranial to caudal direction along the mid-line of the dorsal surface of the mandible, ending at the articulation of the mandible with the quadrate. |
| Caudal mandible | Semi-landmark | Starting on the lateral surface of the caudal mandible adjacent to the cotyla caudalis mandibulae, sampling the outer edge of the mandible in a lateral to medial direction, ending at the medial process. |

**Figure 2 - Source data 2. Descriptions of mandible landmarks and semi-landmarks (following Bjarnason and Benson, 2021).**
