# Supplementary material for: Earliest evidence for fruit consumption and potential seed dispersal by birds
Source: eLife. 2022 Aug 16;11:e74751. doi: 10.7554/eLife.74751 (PMC9381037; doi:10.7554/eLife.74751)
Supplement: Figure 4—source data 1. [file elife-74751-fig4-data1.docx]

| **Taxa** | **Institution** | **Specimen number** |
| --- | --- | --- |
| *Manucodia comrii* | Natural History Museum, London, UK | nhmuk:zoo:A/1905.9.18.8 |
| *Ectopistes migratorius* | Museum of Zoology, The University of Michigan | ummz:birds:68739 |
| *Geospiza fuliginosa* | Natural History Museum, London, UK | nhmuk:zoo:A/1975.15.25 |
| *Pedionomus torquatus* | Natural History Museum, London, UK | nhmuk:zoo:A/1970.12.1 |
| *Conuropsis carolinensis* | Museum of Zoology, The University of Michigan | ummz:birds:68741 |
| *Calcarius lapponicus* | Natural History Museum, London, UK | nhmuk:zoo:A2007.4.1 |
| *Thinocorus rumicivorus* | Natural History Museum, London, UK | nhmuk:zoo:A2004.8.14 |
| *Bombycilla garrulus* | Natural History Museum, London, UK | nhmuk:zoo:A1996.8.1 |

**Figure 4 - Source data 1. Specimens used in the alimentary content analyses.**
